# Effect of a Phytogenic Feed Additive on Growth Performance, Nutrient Digestion, and Immune Response in Broiler-Fed Diets with Two Different Levels of Crude Protein

**DOI:** 10.3390/ani11030775

**Published:** 2021-03-11

**Authors:** Jinquan Wang, Shengchen Su, Chasity Pender, Raj Murugesan, Basharat Syed, Woo Kyun Kim

**Affiliations:** 1 Department of Poultry Science, University of Georgia, Athens, GA 30602, USA; jq.wang@uga.edu (J.W.); su1@uga.edu (S.S.); 2 BIOMIN America Inc., Overland Park, KS 66210, USA; chasity.pender@biomin.net (C.P.); raj.murugesan@biomin.net (R.M.); 3 BIOMIN Holding GmbH, 3130 Getzersdorf, Austria; basharat.syed@biomin.net

**Keywords:** broiler, dietary protein level, phytogenic feed additive, growth performance, immune response

## Abstract

**Simple Summary:**

The rising concerns on antibiotics resistance from using antibiotics in animal production has resulted in an increase in researches on antibiotic alternatives. A phytogenic feed additive from a blend of extracts of oregano, cinnamon, citrus peel, and fructooligosaccharides was evaluated in the present study. The objective of the present study is not only to evaluate the effect of phytogenic feed additive on broiler performance, but also to explore the potential mode of actions through immune response, digestive enzyme activities, nutrient transporter gene expressions and nutrient digestibility. Supplementation of phytogenic feed additives improved broiler FCR through stimulating ileum immunity.

**Abstract:**

The aim of this experiment was to evaluate the effect of a phytogenic feed additive (PFA) on growth performance and nutrient digestibility of broilers fed corn and soybean meal-based diets containing two different levels of crude protein. A 2 × 2 completely randomized factorial arrangement (eight replicates/treatment, 30 birds/replicate) was conducted with a positive control (PC) and negative control (NC) containing crude protein at standard or reduced by 1.5% (equivalent to a reduction of 15 g/kg), respectively, and supplementation of PFA at 0 or 125 ppm of diet. There were no significant interactions found between PFA and CP levels in the current study. Main effect analysis showed that during 0–42 d of age NC diets decreased body weight gain (*p* < 0.05), but increased feed intake (*p* < 0.05) and feed conversion ratio (FCR, *p* < 0.01), whereas supplementation of PFA resulted in a lower FCR (*p* < 0.01). The ileal nutrient digestibility was reduced (*p* < 0.05) in the broilers fed a reduced protein diet at 21 d compared to the standard protein level group, but there were no effects for PFA levels. Similarly, supplementing PFAs showed no effects on digestive enzyme (Alkaline phosphatase, amylase, and lipase) activity in jejunal digesta and jejunal brush border enzyme (maltase, sucrase, and aminopeptidase) activity. Supplementation of PFA downregulated (*p* < 0.05) the mRNA expressions of cytochrome P450 1A and interleukin 6 in the ileum but had no effects on nutrient transporter genes in the jejunum. In conclusion, supplementation of PFA reduced broiler FCR during the whole grow-out period and positively regulated the immune responses in the ileum.

## 1. Introduction

The ban of using antibiotics as growth promoters have spurred research into using plant-derived compounds named phytogenic feed additives (PFAs). The use of PFAs, categorized as sensory and flavoring compounds by European Union legislation (EC 1831/2003), from herbs or spices as antibiotic alternatives are generally recognized as safe [1]. The botanical constituents used in broiler diets as a single compound or multiple cocktails exhibit growth-promoting, immune-regulatory, antimicrobial, stimulating nutrient digestibility and antioxidant properties [2,3,4,5,6].

Dietary protein is a major contributing factor in driving feed cost. Reducing the crude protein level in the broiler diet has recently attracted much attention since it could reduce the feed cost and nitrogen excretion [7,8]. The previous study demonstrated the inclusion of PFA stimulated the nutrient digestibility and small intestine villus height of broilers fed a corn soybean diet [4], however, it remains unknown for the effects of PFA in a low protein diet, and there might be an interaction between PFA and crude protein level.

The PFA in the present study is a blend of extracts of oregano, cinnamon, and citrus peel, and fructooligosaccharides, which has been reported to have a positive result in the performance or metabolism of turkey poults, piglets, aquatic animals, and dairy calves [9,10,11,12]. In broilers, PFA in a diet with reduced metabolizable energy and crude protein reduced plasma cholesterol and improved plasma and meat total antioxidant capacity, gut microbiota, Toll-like signaling molecules, and tight junction genes of broilers [13,14]. Furthermore, it has been reported that PFA also improved the immune system and gut health of broilers infected with *Clostridium perfringens* [15]. In a recent study, supplementation of PFA showed a trend towards improving the livability and performance of broilers challenged with *Eimeria tenella* [16]. Whether PFA can affect nutrient digestion and the immune response of broilers fed a reduced protein diet are unclear.

It is hypothesized that PFA can beneficially modulate anabolism and immunity in broilers fed a low protein diet. The objective of the present study is to evaluate the effects of PFA and dietary protein levels on growth performance, nutrient digestibility and transportation, and the immunity of broilers.

## 2. Materials and Methods

The experimental protocol was reviewed and approved by the University of Georgia Institutional Animal Care and Use Committee (A2019 08-022).

### 2.1. Experimental Treatments

The experiment is a 2 × 2 completely randomized factorial arrangement with two levels of dietary crude protein, standard protein (positive control, PC) following Cobb500 Performance and Nutritional Guide [17] or 1.5% reduction (negative control, NC) and inclusion of PFA (Digestarom^®^ BIOMIN Holding GmbH, Getzersdorf, Austria) at 125 ppm or not. The experimental diets were iso-caloric and formulated based on corn and soybean meal. Broilers were fed throughout a 42-day production period, and no antibiotic growth promoters were used in the diet. PC and NC diets (Table 1) were mixed using a horizontal mixer (Davis Double Ribbon Mixer, Bonner Springs, KS, USA) for 12 min. Then PFA was mixed with 5 kg of either PC or NC diet to create a premix before adding to the mixer for the final treatment diets. All the diets were fed as a mash form. Birds were fed starter, grower, and finisher diet from 0–14, 15–28, and 29–42 d of age, respectively.

### 2.2. Birds Husbandry and Sample Collection

A total of 960 Cobb500 male broiler chicks were obtained on the day of the hatch from a hatchery at Cleveland, GA and randomly allocated into an environmentally controlled house with 32-floor pens (length, 1.52 m; width, 1.22 m; height, 0.61 m) with 30 birds each located at the Poultry Research Center of University of Georgia. All birds were individually weighed and grouped prior to the allocation to ensure an equal initial bodyweight for all pens. The birds were managed as described previously by Wang et al. (2020) [18]. Feed and body weight were measured weekly to determine body weight gain (BWG), feed intake (FI), and feed conversion ratio (FCR). On 21 and 42 d, 10 birds from each pen (replicate) were randomly selected and euthanized by cervical dislocation to collect ileal digesta (obtained from the Meckel’s diverticulum to 1 cm before the ileal–cecal junction) for nutrient digestibility. Jejunum and ileum without contents (rinsed out using phosphate buffer saline) from one bird per pen in treatments PC and PC + PFA at 125 ppm were collected and stored in −80 °C for mRNA expression analysis. The rinsed jejunal digesta were collected in centrifuge tubes and the brush border enzymes were gently scrubbed from rinsed jejunum using a microscope slide. Jejunal digesta and brush border mucous were collected at 21 and 42 d for digestive enzyme activity. Soluble proteins from jejunal digesta and brush border mucous were extracted with 0.01 M PBS at pH 7.2. The samples were centrifuged at 4 ℃, and an aliquot of the supernatant was used for future analyses. Soluble protein was determined by the Braford method using Bio-Rad protein assay kits (BioRad, Hercules, CA, USA).

### 2.3. Chemical Analysis

Chromic (III) oxide (Sigma-Aldrich., St. Louis, MO, USA) was used as a marker for nutrient digestibility in the diet and ileal digesta were analyzed following the method by Williams et al. (1962) [19]. Nitrogen content in feed and digesta was determined using the LECO system as indicated by AOAC International [20] performed at the Agricultural Experimental Station Chemical Laboratories, University of Missouri. Gross energy values in feed and digesta were determined using a calorimeter (IKA C1 Compact Bomb Calorimeter, IKA-Werke., Staufen, Germany).

Calculation:

Nutrient digestibility was calculated using the following equation:

Nutrient digestibility = [1 − (Ci/Co) × (No/Ni)] × 100

where: Ci is the concentration of chromium in the diet; Co is the concentration of chromium in the ileal digesta or feces; Ni is the concentration of the nutrient in the diet; No is the concentration of the nutrient in the ileal digesta or feces; all values were expressed as a percentage of dry matter.

### 2.4. Isolation of mRNA and RT-qPCR

Total mRNA was extracted from jejunal and ileal tissue using Trizol reagent (Invitrogen, Thermo Fisher Scientific, Pittsburgh, PA, USA) according to the manufacturer’s instruction. RNA quantity and purity were determined using a Nanodrop 1000 spectrophotometer (Thermo Fisher Scientific). The cDNA was synthesized from total RNA (1000 ng) using the high-capacity cDNA reverse transcription kit (Thermo Fisher Scientific) and was diluted to 5 ng/µL for RT-qPCR (Real-time polymerase chain reaction) analysis.

Jejunal samples were used to detect nutrient transporter genes including excitatory amino acid transporters (Eaat3), peptide transporter 1 (Pept1), glucose transporter 5 (Glut5) and sodium-glucose transporter 1 (Sglt1) and ileal samples for interleukin (IL) 6, IL-8, heme oxygenase-1 (HO-1), cytochrome P450 isoform 1A1 (CYP1A1), and UDP-glucuronosyltransferases isoform 1A1 (UGT1A1) were analyzed in ileum samples. Glyceraldehyde 3-phosphate dehydrogenase was chosen as a reference gene. The information of primers is shown in Table 2. qPCR was performed on an Applied Biosystems StepOnePlus™ (Thermo Fisher Scientific, Waltham, MA, USA) with iTaq™ Universal SYBR Green Supermix (BioRad, Hercules, CA, USA) using the following conditions for all genes: 95 °C for 10 min followed by 40 cycles of 95 °C for 15 s and 60 °C for 60 s. Samples were run in duplicate and relative gene expression data were analyzed using the 2−ΔΔCt method [21]. The mean ΔCt of PC was used to calculate the ΔΔCt value.

### 2.5. Enzyme Activity Assay

Alkaline phosphatase activity in jejunal digesta is determined using an alkaline phosphatase assay kit (ab83369, Abcam, Cambridge, MA, USA). Alkaline phosphatase activity is reported as U/mg of protein. One unit of alkaline phosphatase is defined as the amount of enzyme causing the hydrolysis of one micromole of para-Nitrophenylphosphate per minute at pH 9.6 and 25 °C. Amylase activity in jejunal digesta is measured using EnzChek Ultra Amylase Assay Kit (E33651, Molecular Probes, Eugene, OR, USA). Amylase activity is reported as U/mg of protein. One unit of amylase is the amount of enzyme will generate 1 micromole of glucose from corn starch. Amylase from Bacillus sp. was used as the standard. Lipase activity in jejunal digesta is determined using Lipase Activity Assay Kit (MAK047, Sigma-Aldrich, St. Louis, MO, USA) and is reported as mU/mg of protein. One unit of lipase is the amount of enzyme that will generate 1.0 µmole of TNB per minute at 42 °C.

Jejunal brush border enzyme activities (maltase, sucrase and leucine aminopeptidase) were analyzed. For maltase and sucrase, jejunal mucous were homogenized in 100 mM mannitol 2 mM HEPES/KOH (pH 6.5), then centrifuged at 2200× *g* for 10 min. Aliquots of the supernatant were stored at −20 °C. Maltase and sucrase activities were analyzed according to Dahlqvist methods [22] with modification for microplate (Corning Costar 3631, Corning, NY, USA) assay at 42 °C for 20 min incubation. Sucrase or maltase unit were described as mg of glucose generated from sucrose or maltose per minute per mg of protein. The background glucose content from maltose and sucrose was measured and deducted from the reading. For leucine aminopeptidase, 15 µL of undiluted homogenized tissue was incubated with 135 µL 1 µmole L-leucine-p-nitroanilide 42 °C per 0.01 mole of PBS for 30 min. We used 4-nitroaniline (Fisher AC18069-1000, Hampton, NH, USA) as a standard. Change of absorbance was detected on a Molecular Devices microplate reader at O. D. 405 mM [23] (Sun, 2007). One unit of aminopeptidase N is defined as the hydrolysis of 1 µmol of the substrate in one minute at 42 °C, pH 7.0.

### 2.6. Statistical Analysis

Growth performance and nutrient digestibility data were analyzed using a two-way ANOVA model and gene expression and enzyme activity data were analyzed using a one-way ANOVA model as a completely randomized design using the GLM procedure of SAS 9.4 [24]. Significant level was set at *p* < 0.05 and tendency at 0.05 ≤ *p* < 0.10. Each pen was regarded as an experimental unit. The least square means were reported in the results. Treatment means were further separated using Tukey’s multiple range test when the interaction is presented.

## 3. Results and Discussions

Because there were no significant interactions (*p* > 0.1) found in the current study, our results and discussion were focused on the main effect of crude protein and PFA on broiler performance, nutrient digestibility, and gene expression of nutrient transporter and immunity.

### 3.1. Growth Performance

Birds in all the pens maintained general health throughout the trial. The effect of crude protein level and phytogenic feed additive on the growth performance of broilers is shown in Table 3. Main effects showed that diets with low crude protein level had trended to decrease (*p* = 0.076) BWG and increased (*p* = 0.089) FI, but FCR was significantly increased during 0–21 (*p* = 0.032) and 0–42 d of age (*p* < 0.01). Supplementation of PFA reduced FCR during 0–21 d (*p* = 0.042) and the whole grow out period (*p* = 0.034). There were no interactions between dietary protein and PFA on the growth performance.

In the present study, reducing the dietary protein at 1.5% showed a negative effect on growth by decreasing BWG and increasing FCR and FI, particularly in the finisher phase. Dietary protein is potentially used for muscle growth and immunity in animals [25]. Broilers in the present study attempted to increase FI to meet their protein and amino acid requirements for growth and maintenance needs. Whereas a reduced protein diet could be insufficient in supporting both roles even with the increased FI. It is plausible that PFA in the diet may reduce the immunity cost on energy and protein [4]. The nutrients are redirected to animal growth thus broilers fed diet contain PFA had a better FCR. Further studies are necessary on the mechanism of PFA on broiler immunity and performance. Studies reported that reduction of crude protein by 1.7% decreased BWG while the FCR maintained the same [26], but a 2% reduction in a miscellaneous meal diet reduced both FI and BWG in broilers [5]. Dietary protein level was reported as a significant positive contributor for broiler BWG and feed efficiency by Pesti. (2009) from summarizing 26 types of research [27], however, the protein quality from the ingredients, reduction level and rearing environments are played as cofactors on the dietary protein level and broiler performance.

The PFA is a blend of citrus peel, cinnamon, oregano and fructooligosaccharides. During the finisher and whole grow-out period in the present study, the increased FCR was induced by reduced dietary protein, whereas PFA supplementation improved the broiler efficiency This is in agreement with the previous study that supplementation of PFA improved performance and offset the negative effect of a low protein diet [28]. Sadek et al. (2014) and Murugesan et al. (2015) reported PFA improved the feed efficiency of broilers [4,29]. Paraskeuas et al. (2016) found that the reduction of dietary protein by 1.32% and ME by 0.8 MJ/kg increased FCR, but PFA addition did not affect the growth performance of broilers [13]. The response of phytogenic compounds on broiler performance may be contributed by variable sources of plants and processing methods of extracting those active compounds. The recent studies are more likely to show the beneficial effects on growth performance, which may be due to the improvement in extraction and refinement of those active compounds [12,13,14].

### 3.2. Nutrient Digestibility

The effect of crude protein level and phytogenic feed additive on the growth performance of broilers is shown in Table 4. Low dietary protein diets decreased (*p* = 0.047) crude protein digestibility at 21 d (Table 4), but PFA addition did not cause significant differences in the digestibility of dry matter, crude protein, and ileal digestible energy among the treatments at 21 and 42 d of age in the present study.

The activities of digestive enzymes, including amylase, lipase, aminopeptidase, sucrose, maltase, and alkaline phosphatase were not different at 21 and 42 d in the present study (Table 5).

In the present study, the decreased protein digestibility in the low protein diet negatively affected BWG and FCR, while supplementation of PFA at 125 mg/kg had no effect on digestibility. Zumbaugh et al. (2020) [12] reported that inclusion of a similar blend of PFA at 1 g/kg in the diet with a 1.5% reduction of dietary protein improved BWG, but did not affect FCR, protein intake, and protein digestibility of turkey poults. Meanwhile, there are studies that reported feeding PFAs to broilers improved crude protein digestibility and AMEn [4,30,31]. The different results of feeding PFAs on poultry species may associate with mechanisms of PFAs. The PFAs generally affect broilers through the mechanism of anti-oxidation, stimulating digestive enzymes, or regulating the immune response. Additionally, there was no effect of PFA on digestive enzymes for broilers fed a standard protein level diet in the present study, and similar results were found when turkey poults were fed PFA except a reduction of aminopeptidase activity [12]. The different results in nutrient digestibility and digestive enzymes could be related to the PFA dose and poultry species, which deserves further study.

### 3.3. Gene Expression of Transporter and Immunity

The present study further detected the mRNA profiles of nutrient transporters and immune parameters in treatments PC and PFA at 125 ppm (Table 6). In contrast with PC, the mRNA expressions of Eaat3, Pept1, Glut5, and Sglt1 in the jejunum were not influenced by supplementing PFA at 21 and 42 d of age. Ileal immune parameters CYP1A1 (*p* = 0.030) and IL-6 (*p* = 0.037) were downregulated by PFA at 21 d, but not for IL-8, HO-1, UGA1A1 at 21 and 42 d.

The unaffected transporters in broilers fed the PFA diet in the present study, coupled with unchanged digestive enzyme activity and most nonsignificant digestibility parameters, indicate that PFA as a phytogenic nutraceutical is not enough to trigger significant differences in nutrient digestion, transportation, and assimilation for broilers. Likely, in turkeys, these transporters were not influenced by the PFA diet [12].

Importantly, in the present study, PFA deregulated ileal CYP1A1 and IL-6, indicating that PFA may reduce inflammation and redirect nutrients towards growth in broilers. Expression of CYP1A1 is a sensitive indicator for certain immune cell loss and susceptibility to enteric infection [32,33]. IL-6 acts as a pro-inflammatory cytokine especially in the smooth muscle cell [34]. PFA protected the intestinal barrier by upregulating the tight junction protein gene in broilers [14]. The beneficial immune response and intestinal barrier may be the main contributors for the improvement in feed efficiency by PFA. It is known that immune response cost energy and protein [35], thus, the down-regulation of immunity genes in the present study may partially explain the improved FCR with PFA addition. Due to the less demand on immunity, more nutrients are possibly redirected to growth. Recent studies found that phytochemicals modulated intestinal endogenous bactericidal peptides and muscle physicochemical property [36,37], and whether PFA has an effect on these aspects needs further study.

## 4. Conclusions

In conclusion, dietary supplementation of PFA improved feed conversion ratio and ileum immune gene expression in the present study. However, the nutrient digestibility, nutrient transporter gene expression, and digestive enzyme activities were not influenced for broiler-fed diet supplemented with PFAs. The beneficial effects of PFA on broiler performance could be linked with the antioxidant and anti-inflammatory compounds from PFA-related plants.

## Figures and Tables

**Table 1:** Feed formulation and composition of the experimental diets.

Item	0–14 d		15–28 d		29–42 d	
	PC ^1^	NC ^1,2^	PC ^1^	NC ^1,2^	PC ^1^	NC ^1,2^
Ingredient (%)						
Corn	58.17	58.20	62.75	62.25	64.52	69.00
Soybean meal	36.42	33.27	31.58	28.21	29.36	25.49
DCP ^1^	1.57	1.57	1.44	1.47	1.24	1.26
Soybean oil	1.70	2.56	2.19	2.54	3.00	2.35
Limestone	1.18	1.18	1.13	1.14	1.05	1.07
Common salt	0.30	0.30	0.30	0.30	0.30	0.30
DL-methionine	0.25	0.24	0.22	0.19	0.18	0.16
Premix ^1,3^	0.25	0.25	0.25	0.25	0.25	0.25
L-lysine-HCL	0.08	0.09	0.08	0.09	0.02	0.05
Sand	0	2.21	0	1.48	0	0
Calculated nutrient ^1^ (%)						
ME ^1^ (kcal/kg)	3008	3008	3086	3086	3160	3160
Crude protein	22.00	20.50	20.00	18.50	19.00	17.50
Dig-Lysine	1.18	1.10	1.05	0.97	0.95	0.88
Dig-Methionine	0.58	0.55	0.52	0.48	0.47	0.43
Dig-TSAA	0.88	0.83	0.80	0.74	0.74	0.68
Dig-Threonine	0.78	0.73	0.71	0.65	0.67	0.62
Ca	0.90	0.90	0.84	0.84	0.76	0.76
Non-phytate P	0.45	0.45	0.42	0.42	0.38	0.38
Analyzed nutrient						
Crude protein, %	22.24	20.81	20.36	18.25	19.15	17.88
GE ^1^, kcal/kg	3815	3803	3986	3957	4016	4004

^1^ DCP, dicalcium phosphate; ME, metabolizable energy; TSAA, total sulfur amino acid; PC, positive control containing standard crude protein; NC, negative control containing reduced crude protein by 1.5% (equivalent to a reduction of 15 g/kg), GE, gross energy.^2^ The amino acids ratio to lysine remained the same as positive control. ^3^ Provided per kg of diet: vitamin A (retinyl acetate), 8000 IU; cholecalciferol, 1000 IU; vitamin E (DL-tocopheryl acetate), 20 IU; vitamin K, 0.5 mg; thiamin, 2.0 mg; riboflavin, 8.0 mg; d-pantothenic acid, 10 mg; niacin, 35 mg; pyridoxine, 3.5 mg; biotin, 0.18 mg; folic acid, 0.55 mg; vitamin B12, 0.010 mg; manganese, 120 mg; iodine, 0.70 mg; iron, 100 mg; copper, 8 mg; zinc, 100 mg; and selenium, 0.30 mg.

**Table 2:** Information of primers for quantitative real-time PCR.

Gene Name	Primer (5′→3′)		Length	Reference Sequence
	Forward	Reverse		
Nutrient transporters				
Eaat3	tgctgctttggattcagtgt	agcaatgactgtagtgcagaagtaatatatg	79	XM_424930.5
Pept1	cccctgaggaggatcactggtt	caaaagagcagcagcaacga	66	NM_204365.1
Glut5	ttgctggctttgggttgtg	ggaggttgagggccaaagtc	60	XM_417596.5
Sglt1	gccgtggccagggctta	caataacctgatctgtgcaccagta	71	NM_001293240.1
Immunity				
CYP1A1	gcttcaaccccaacagctac	gtgttcatgttcaccacgct	118	NM_205147.1
IL-6	ataaatcccgatgaagtgg	ctcacggtcttctccataaa	146	NM_204628.1
IL-8	cgttcagcgattgaactccg	ctgccttgtccagaattgcc	211	NM_205018.1
HO-1	cacgagttcaagctggtcac	ctgcagctccatcggaaaat	120	NM_205344.1
UGT1A1	ccaacctgccaaagaacgtg	ccctcgtaaacaccgtgtga	115	XM_015289249.1
GAPDH	tcagcagcaggcttcactac	gctaaggctgtggggaaagt	161	NM_204305.1

CYP1A1, cytochrome P450 family 1 subfamily A member 1; Eaat3, excitatory amino acid transporter 3; GAPDH, glyceraldehyde 3-phosphate dehydrogenase; Glut5, fructose transporter 5; HO-1, heme oxygenase 1; IL, interleukin; Pept1, peptide transporter 1; SEM, standard error of the mean; Sglt1, sodium glucose linked transporter 1; UGT1A1, UDP-glucuronosyltransferases isoform 1A1.

**Table 3:** Effects of crude protein level and phytogenic feed additive on the growth performance of broilers.

Item	BWG (g/bird)			FI (g/bird)			FCR		
	0–14 d	0–28 d	0–42 d	0–14 d	0–28 d	0–42 d	0–14 d	0–28 d	0–42 d
Main effect of dietary protein level									
PC ^1,2^	324	1235	2488	457	1805	4110	1.409	1.420 ^B^	1.665 ^B^
NC ^1,2^	322	1207	2424	460	1882	4194	1.430	1.510 ^A^	1.731 ^A^
SEM ^2^	2.91	14.63	22.03	4.65	28.5	35.7	0.018	0.021	0.010
Main effect of PFA level									
0	323	1223	2434	458	1838	4185	1.425	1.479 ^A^	1.724 ^A^
125 ppm	324	1218	2478	459	1856	4157	1.420	1.456 ^B^	1.683 ^B^
SEM ^2^	2.97	14.1	24.5	4.53	31.28	32.3	0.020	0.016	0.013
Treatments									
PC ^1,2^ + 0	321	1235	2479	452	1777	4085	1.411	1.390	1.668
NC ^1,2^ + 0	328	1212	2498	461	1885	4204	1.427	1.506	1.760
PC ^1,2^ + PFA ^2^	325	1235	2390	463	1833	4127	1.432	1.444	1.662
NC ^1,2^ + PFA ^2^	320	1202	2458	457	1879	4184	1.370	1.515	1.702
SEM ^2^	2.38	10.5	18.0	2.99	22.5	23.9	0.018	0.021	0.010
*p*-value									
Protein level	0.639	0.211	0.076	0.637	0.108	0.089	0.475	0.032	<0.010
PFA ^2^	0.808	0.820	0.221	0.911	0.702	0.984	0.984	0.042	0.034
Interaction	0.170	0.821	0.484	0.294	0.500	0.525	0.885	0.584	0.104

Means within a column with different superscripts differ: ^A,B^ *p* < 0.05. ^1^ standard crude protein levels were 22.0, 20.0 and 19.0% at 0–14, 15–28 and 29–42 d of age, respectively; reduced protein group were reduced crude protein by 1.5%. ^2^ PC, positive control; NC, negative control; PFA, phytogenic feed additive, Digestarom^®^ P.E.P.at 125 ppm; SEM, standard error of the mean with *n* = 8.

**Table 4:** Effects of crude protein level and phytogenic feed additive on the nutrient digestibility of broilers.

Item	DM (%)		CP (%)		IDE (kcal/kg)	
	21 d	42 d	21 d	42 d	21 d	42 d
The main effect of dietary protein level						
PC ^1,2^	71.8	71.1	81.2 ^A^	79.7	2837	2878
NC ^1,2^	70.6	72.1	78.2 ^B^	78.0	2791	2838
SEM ^2^	1.13	0.75	0.96	0.89	51.0	34.1
The main effect of PFA level						
0	71.0	71.6	79.6	78.9	2807	2853
125 ppm	71.4	71.6	79.8	78.8	2821	2863
SEM ^2^	1.12	0.76	1.03	0.95	50.6	34.3
Treatments						
PC ^1,2^ + 0	71.2	71.4	80.9	80.0	2834	2849
NC ^1,2^ + 0	70.9	71.9	78.4	77.9	2781	2857
PC ^1,2^ + PFA ^2^	72.4	70.9	81.4	79.4	2840	2826
NC ^1,2^ + PFA ^2^	70.4	72.3	78.1	78.2	2801	2899
*p*-value						
PFA	0.827	0.997	0.910	0.925	0.8596	0.846
CP level	0.476	0.389	0.047	0.226	0.5432	0.426
Interaction	0.622	0.666	0.789	0.761	0.9296	0.516

Means within a column with different superscripts differ: ^A,B^ *p* < 0.05. ^1^ standard crude protein levels were 22.0, 20.0 and 19.0% at 0–14, 15–28 and 29–42 d of age, respectively. ^2^ PC, positive control; NC, negative control; DM, dry matter; IDE, ileal digestible energy; PFA, phytogenic feed additive, Digestarom^®^ P.E.P. at 125 ppm; SEM, standard error of the mean with *n* = 8.

**Table 5:** Effect of phytogenic feed additive (PFA) on the jejunal digesta and brush border enzyme activities of broilers.

Item	Jejunal Digesta			Brush Border Enzyme		
	**Alkaline Phosphatase U/mg**	**Amylase U/mg**	**Lipase mU/mg**	**Maltase mg/mg**	**Sucrase mg/mg**	**Aminopeptidase µmol/mg**
21 d of age						
Control ^2^	1.549	4.912	23.172	3.236	2.458	0.202
PFA ^1^	1.109	4.348	22.963	4.161	2.456	0.195
SEM ^1^	0.291	0.158	1.564	0.25	0.15	0.009
*p*-value	0.289	0.475	0.950	0.115	0.996	0.693
42 d of age						
Control ^2^	1.381	4.764	22.560	2.983	2.011	0.098
PFA ^1^	1.048	4.489	17.500	2.769	2.077	0.101
SEM ^1^	0.231	0.161	2.246	0.33	0.38	0.011
*p*-value	0.602	0.8812	0.275	0.659	0.845	0.485

^1^ PFA, phytogenic feed additive, Digestarom^®^ P.E.P. added at 125 ppm of diet; SEM, standard error of the mean with *n* = 8. ^2^ Containing standard crude protein levels were 22.0, 20.0 and 19.0% at 0–14, 15–28 and 29–42 d of age, respectively.

**Table 6:** Effect of PFA on the gene expression of nutrient transporters and immune parameters of broilers.

Item	Nutrient Transporter				Immunity				
	Eaat3 ^1^	Pept1 ^1^	Glut5 ^1^	Sglt1 ^1^	CYP1A ^1^	IL-6 ^1^	IL-8 ^1^	HO-1 ^1^	UGAT1A1 ^1^
21 d of age									
Control ^2^	1.09	1.04	1.07	1.04	1.42 ^A^	1.16 ^A^	1.11	1.01	1.03
PFA ^1^	0.73	0.71	0.92	0.86	0.38 ^B^	0.57 ^B^	1.19	0.99	0.86
SEM ^1^	0.12	0.12	0.17	0.14	0.25	0.15	0.26	0.09	0.1
*p*-value	0.193	0.151	0.721	0.583	0.032	0.037	0.507	0.881	0.279
42 d of age									
Control ^2^	1.18	1.2	1.14	1.24	0.91	1.34	1.22	1.03	1.02
PFA ^1^	0.82	0.75	0.83	0.78	1.03	1.04	1.35	1.07	1.1
SEM ^1^	0.22	0.23	0.18	0.26	0.33	0.38	0.32	0.12	0.12
*p*-value	0.429	0.119	0.135	0.402	0.945	0.544	0.699	0.821	0.679

^A,B^ means within a column with different superscripts trend to be different (*p* < 0.05). ^1^ Eaat3, excitatory amino acid transporter 3; Pept1, peptide transporter 1; Glut5, fructose transporter 5; Sglt1, sodium glucose linked transporter 1; CYP1A1, cytochrome P450 family 1 subfamily A member 1; HO-1, heme oxygenase 1; IL, interleukin; UGT1A1, UDP-glucuronosyltransferases isoform 1A1; PFA, phytogenic feed additive, Digestarom^®^ P.E.P. added at 125 ppm of diet; SEM, standard error of the mean with *n* = 8. ^2^ Containing standard crude protein levels were 22.0, 20.0 and 19.0% at 0–14, 15–28 and 29–42 d of age, respectively.

## Data Availability

Data available on request due to privacy restrictions.
